# Personal

**Published:** 1911-09

**Authors:** 


### PERSONAL

Dr. Charles W. Bethune of Buffalo has removed his office from 262 Niagara Street, to the Professional Building, 131 Allen Street. Hours 1 to 3 and 6 to 8. Sundays, 2 to 5. Telephone, Tupper, 1251-W.

Dr. J. E. Walker, of the Steuben Sanitarium at Hornell, N. Y., has returned from his vacation and taken up his position again as superintendent much to the pleasure of the guests and everyone connected with the institution.

---

Dr. Benjamin H. Grove announces that he has occupied his new suite of offices at 344 Pearl St. Hours 9 a. m. to 12.30 P. M. Both 'phones.

---

Dr. Frank A. Love spent part of August on Georgian Bay.

---

Dr. Dewitt H. Sherman has recently returned from Newport.

---

Dr. Joseph T. Cook has returned from the Adirondacks.

---

Dr. Richard H. Satterlee had an unusual experience at the recent burning of the Hotel Frontenac. After the fire, he went to South Butler and has just returned to Buffalo.

---

Dr. H. W. Wiley took a few day's rest at Niagara Falls, late in August.

---

Dr. Ida C. Bender has returned from her summer home in Massachusetts.

---

Dr. Maud J. Frye of Buffalo, sailed in July for a three months' stay in Europe.

---

Dr. and Mrs. G. A. Himmelsbach of Buffalo, sailed early in August for Europe.

---

Dr. Julius Richter of Buffalo, is spending a month at the Mayo Clinics in Rochester, Minn.

---

Dr. Charles A. Bentz is in Europe for three months' study.

---

Dr. Francis M. O'Gorman of Jefferson street, returned from a two-weeks trip up the lakes late in August.

---

Health Commissioner Fronczak has received an invitation to address the International Municipal Congress and Exposition in Chicago during its session, September 18th to 30th, on the subjects of milk supply, school inspection and garbage disposal.

